# Prevalence and Genetic Analysis of Isoniazid-Resistant Tuberculosis in Eastern Uttar Pradesh, India

**DOI:** 10.7759/cureus.80243

**Published:** 2025-03-08

**Authors:** Nandini Singh, Amresh K Singh, Sarita Sarita, Sushil Kumar, Ashwini K Mishra, Narendra P Singh

**Affiliations:** 1 Zoology, Deen Dayal Upadhyaya Gorakhpur University, Gorakhpur, IND; 2 Microbiology, Baba Raghav Das Medical College, Gorakhpur, IND; 3 Respiratory Medicine, Baba Raghav Das Medical College, Gorakhpur, IND

**Keywords:** drug resistance tuberculosis, genotype® mtbdrplus assay, high- and low-level resistance, inha, isoniazid, katg

## Abstract

Background

Tuberculosis (TB) is an airborne bacterial infection caused by *Mycobacterium tuberculosis. *It continues to pose a major threat, India bore the highest TB burden accounting for 27% of global cases in 2022. The emergence of drug resistance, especially primary medications such as isoniazid (INH), hinders treatment and containment efforts. Genetic alterations in the *kat*G and *inh*A genes are the main contributors to INH resistance, resulting in high and low levels of resistance, respectively.

Objectives

This study investigates the genetic mutational patterns of INH resistance in TB cases, focusing on their prevalence and association with demographical, geographical, and clinical features.

Methods

A cross-sectional prospective study was conducted in our tertiary care center located in eastern Uttar Pradesh (UP), India, from June 2022 to May 2024. A total of 6,954 highly suspected TB cases, including pulmonary and extra-pulmonary samples, were evaluated. After fluorescence microscopy, line probe assay (LPA) was used to analyze 1,998 (28.73%) sputum-positive samples for* kat*G and *inh*A mutations, which confer INH resistance.

Results

Among 1,998 sputum-positive samples tested with LPA, valid results were obtained for 1,993 cases. Of these, 131 (6.57%) showed INH resistance, with high-level INH resistance detected in 102 (77.86%) cases, predominantly linked to *kat*G MUT1 (S315T1) mutations. Low-level resistance was identified in 29 (22.14%) cases, primarily associated with *inh*A MUT1 (C-15T) mutations. Among INH resistance cases, females were significantly younger than males (mean age 28.49±14.16 vs. 40.65±16.03; p-value<0.001), but male cases were higher than female (86[65.64%] vs. 45[34.35%]; p-value=0.039). However, age distribution was comparable between high and low-level INH resistance. Geographically, Gorakhpur emerged as a hotspot for high-level resistance 40/102 (39.21%), while Deoria had the highest prevalence of low-level resistance 9/29 (34.48%).

Conclusion

Our study identified the prevalence of *kat*Gmutations in high-level and *inh*A mutations in low-level INH resistance in eastern UP, India. This highlights the need for region-specific public health strategies, including better joint management of comorbidities and enhanced diagnostic capacity to address the burden of drug-resistant TB in this high-prevalence region.

## Introduction

Tuberculosis (TB) is an airborne bacterial disease caused by *Mycobacterium tuberculosis *(*M. tuberculosis*), posing a significant global health threat. In 2022, India accounted for 27% of the world’s TB cases, making it the highest TB burden country [1]. Uttar Pradesh (UP), India’s most populous state, contributes a considerable portion to the country’s total TB cases [2]. Drug-resistant tuberculosis (DR-TB) occurs when *M. tuberculosis* strains become resistant to one or more first-line anti-TB pharmaceuticals [3]. The other forms of TB include multidrug-resistant TB (MDR-TB), in which mycobacterium is resistant to first-line drugs, i.e., rifampicin (RMP) and isoniazid (INH), and extensively drug-resistant TB (XDR-TB), in which mycobacterium is resistant to first-line and second-line drugs, i.e., fluoroquinolones and group A drugs [2,4]. Timely diagnosis and treatment of infectious TB are important in controlling its spread. However, the efficacy of INH, a critical first-line drug for TB treatment, is influenced by *M. tuberculosis* resistance level [5]. INH resistance at a high or low level is associated with mutations in the *kat*G and *inh*A genes. High-level resistance is mostly linked to mutations in the *kat*G gene, while low-level resistance is associated with mutations in the *inh*A promoter region [6]. Such mutations often lead to distinct therapeutic challenges, as high-level INH-resistant strains may also show pan-drug resistance, making MDR-TB treatment regimens difficult.

The GenoType® MTBDRplus assay is a widely employed molecular test known for identifying drug resistance in TB. Often referred to as the first-line line-probe assay (FL-LPA), this test uses fluorescent-tagged peptide arrays to rapidly detect mutations linked to resistance. Its high sensitivity and specificity allow it to pinpoint resistance patterns early, which is vital for accurate treatment. Furthermore, its capacity to simultaneously recognize multiple resistance mechanisms boosts its effectiveness, establishing it as a crucial resource in clinical diagnostics and the study of antimicrobial resistance [7]. It is capable of detecting mutations in the *kat*G gene that are associated with a high level of resistance to INH and mutations in *inh*A promoter region associated with a low level of resistance [8]. The *kat*G gene codes for a catalase-peroxidase enzyme necessary for the activation of INH, and mutations S315T, which prevent activation, lead to high-level resistance. The *inh*A gene, which encodes an enzyme involved in mycolic acid synthesis, mutates in its promoter region (C-15T) resulting in overexpression of the enzyme, thus reducing the binding of the drug, which causes low-level resistance [9].

Geographical assessment is crucial in understanding TB dynamics, as it highlights regional variations in disease prevalence, treatment outcomes, and resistance patterns. These variations often arise from differences in healthcare infrastructure, socioeconomic conditions, and environmental factors, making geographical analysis essential for shaping effective public health interventions and policies [10]. Previous history of TB and comorbidities such as diabetes, HIV, and anemia, as well as tobacco and alcohol use and prior surgeries, are increasingly acknowledged as major risk factors for the onset and worsening of DR-TB. Research indicates that these comorbidities can intensify the severity of TB and complicate treatment outcomes, potentially resulting in higher resistance rates to first-line drugs such as INH [11]. Comprehending the interplay between DR-TB and these factors is essential for developing more effective treatment strategies and for managing complications in patients with multiple health issues.

In this study, we performed the cross-sectional prospective investigation of high- and low-level INH resistance in patients with TB, focusing on the genetic mutational patterns associated with resistance levels. By examining a sample of individuals with varying degrees of INH resistance, we aimed to uncover critical insights into the demographic, clinical, and genetic factors that contribute to INH resistance in TB. Our analysis included comparisons of age, gender, treatment history, and sources of TB infection among patients, with a particular emphasis on identifying significant patterns of genetic mutations linked to INH resistance. Geographical diversity helps to uncover disparities in TB management, healthcare access, and risk factors such as overcrowding or malnutrition, identifying potential hotspots for INH-resistant strains.

## Materials and methods

Inclusion criteria and sample collection

Patients highly suspected of TB were identified based on the National Tuberculosis Elimination Program (NTEP) guidelines, and those with a history of previous TB treatment and treatment failures or defaulters were included in this study [12]. Samples from all age categories were collected from pulmonary and extra-pulmonary sites, i.e., cerebrospinal fluid, pus, and body fluids. TB confirmation was done using fluorescence microscopy (FM). Patients were excluded if they had a low index of suspicion for TB or if their symptoms and risk factors did not strongly indicate TB as a likely diagnosis. Individuals with confirmed TB but negative sputum smears for acid-fast bacilli (AFB) on FM were also excluded. Additionally, cases in which the line probe assay (LPA) produced invalid or inconclusive results, preventing the determination of drug resistance, were not included in the study.

This study was conducted at the Intermediate Reference Laboratory (IRL) of Baba Raghav Das Medical College, Gorakhpur, located in eastern UP, India, from June 3, 2022 to May 20, 2024. In patients who met the inclusion criteria, a total of 6954 specimens were collected from both pulmonary and extra-pulmonary sites. Specimens were transported under a strict cold chain to the IRL, and further samples were processed using the standard protocol by N-acetyl-L-cysteine-NaOH method for decontamination, followed by direct smear microscopy using FM staining for preliminary identification of AFB [13].

Diagnosis and interpretation by GenoType® MTBDRplus 2.0/LPA

DNA Extraction

Genotype® MTBDRplus 2.0 assay was used to establish INH and RMP resistance for DR-TB detection. After specimen collection, the next step was DNA extraction, which involved breaking down the cell walls of the *M. tuberculosis* to release its genetic material. DNA extraction was performed on decontaminated patient samples using a lysis process.

Amplification and Hybridization

The master mixture prepared for amplification consisted of 35 µL of primer nucleotide mix (solution AB-B) and 10 µL of solution AB-A, making a final volume of 45 µL for each sample. Reverse hybridization was performed using an automatic machine (genotype [GT] blot) [12]. Denaturation solution was then added to the amplified sample, immediately followed by pre-warmed hybridization buffer. After adding the strip, the tray was transferred to the GT blot machine. The stringent wash solution, rinse solution, conjugate solution, and substrate were automatically added by the machine in the next steps. Further, the strip was washed out of the tray and dried [13].

Result Interpretation

After the strip was removed from the hybridization process, it was attached to the paper, and the result readings were recorded. The strip was then pasted and stored in a dark environment to protect it from light. The kit included an evaluation/control sheet [14], which was used when the strips were developed. The strips were pasted in the designated fields on the sheet, ensuring proper alignment with the conjugate control and amplification control bands. Each LPA strip contained 27 reaction compartments, including 7 controls [13].

Risk factor analysis

Risk factor data were gathered using a questionnaire form during sample collection and through telephonic conversations. Our study included risk factors such as previous TB history, any surgeries, and ongoing conditions such as diabetes, anemia, etc. We also investigated the sources of TB infection or any connections with TB patients. Some participants could not recall any such sources, so they were classified as having no comorbidities.

Our facility functions as an IRL for TB in eastern UP, India, serving patients referred from seven districts, including Deoria, Basti, Sant Kabir Nagar, Siddharthnagar, Kushinagar, Maharajganj, and Gorakhpur. Our study was permitted by the Institutional Human Ethical Committee (IHEC) of Baba Raghav Das Medical College, Gorakhpur, vide letter no. 11/IHEC/2022, and consent had been taken from all the participants. We collected the data that covered basic demographics and the participant's original hometown or district and workplace.

Statistical analysis

Statistical analysis was performed using SPSS Version 15.0 (SPSS Inc., Chicago). Qualitative variables were expressed as numbers and percentages, while quantitative values were displayed as the mean±standard deviation. All statistical significance was set at p≤0.05. Chi-square test was used for categorical data, while an independent sample t-test was applied to continuous data.

## Results

**Figure 1:**
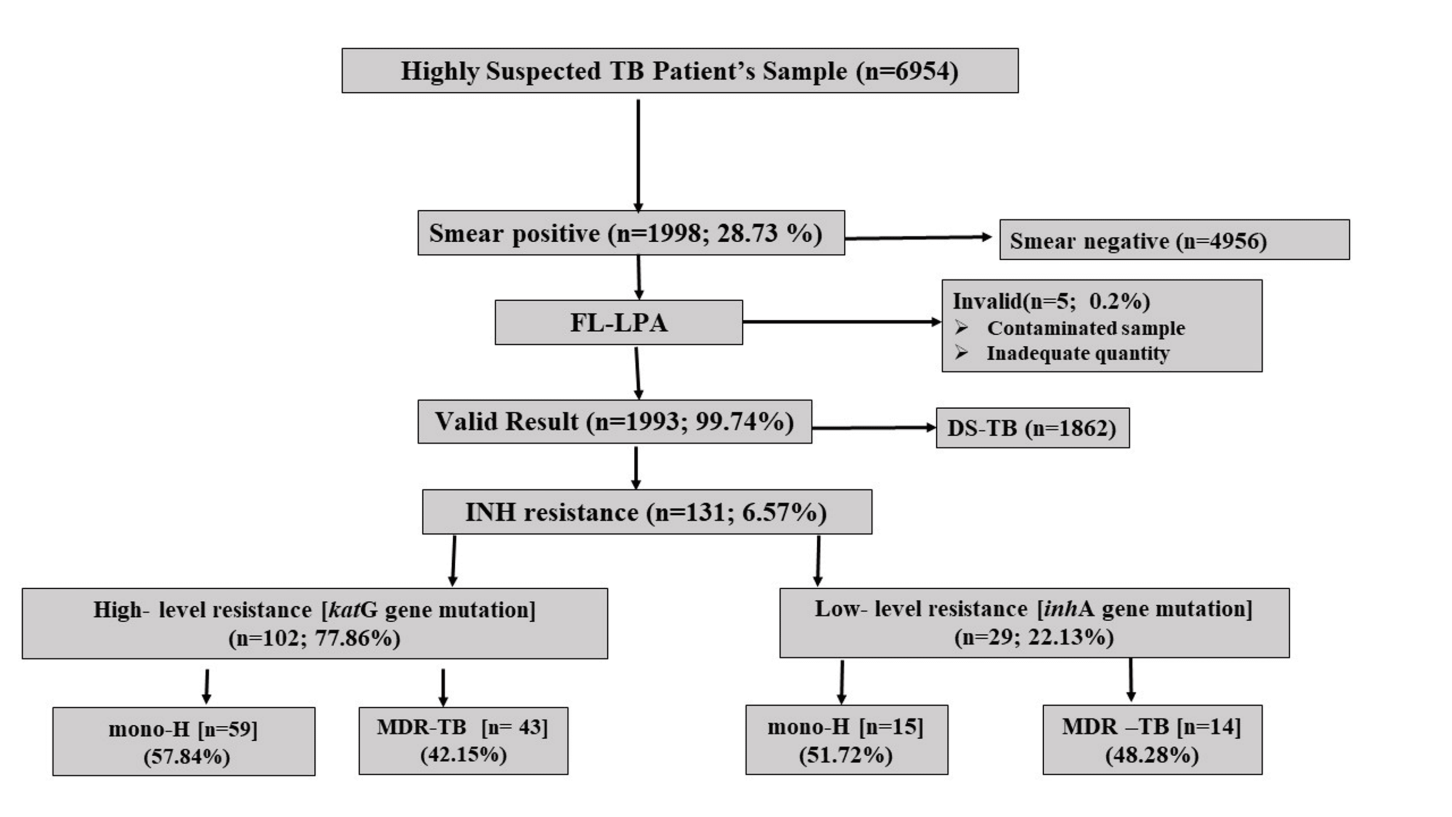
Flow chart of inclusion and exclusion of cases for INH-resistant TB assessment DS-TB: Drug-sensitive tuberculosis; FL-LPA: First-line line-probe assay; INH: Isoniazid; MDR-TB: Multidrug-resistant tuberculosis; Mono-H: INH-monoresistant tuberculosis; TB: Tuberculosis

The sequence of steps for including and excluding cases in the assessment of INH-resistant TB can be found in Figure 1. This study analyzed 6,954 TB samples, of which 1,998 (28.7%) were smear-positive by FM microscopy. Among the smear-positive samples, five (0.3%) gave invalid results due to inadequacy and contamination, and, finally, 1,993 (99.7%) cases were examined using FL-LPA. Of these, 1,862 (93.4%) were classified as drug-sensitive TB (DS-TB), while 131 (6.6%) were identified as INH-resistant cases. Within INH-resistant cases, 102 (77.86%) exhibited high-level INH resistance associated with *kat*G mutations. Among these, 59 (57.84%) were classified as INH-monoresistant TB (mono-H), indicating resistance to INH only, while 43 (42.16%) were MDR-TB, showing resistance to both INH and RMP. However, 29 (22.14%) showed low-level resistance linked to *inh*A mutations, in which 15 (51.72%) cases were identified as mono-H and 14 (48.28%) were classified as MDR-TB.

Demographic characteristics and risk factors of INH-resistant cases

Demographic characteristics stratify TB cases based on the presence or absence of INH resistance and presence of high- and low-level resistance, providing a clear comparison of various factors influencing the distribution and progression of the disease. Key demographic features, such as age, gender, and socioeconomic status, are detailed to show how these variables correlate with INH resistance in TB cases (Table 1). Age was comparable between INH resistance cases and gender distribution also revealed a significantly higher incidence in males (86 [65.64%] vs. 45 [34.35%], p-value=0.039), but age distribution between males and females in INH resistance showed that females were significantly younger than males (28.49±14.16 vs. 40.65±16.03, p-value≤0.001). Age and gender were also comparable between our stratified groups as shown in Table 1.

In this study, a significant proportion of the subjects had previously received TB treatment, which is important for its interpretation, as the proportion of subjects with past treatment was significantly higher in INH resistance (Table 1). This suggests that individuals who have had incomplete or ineffective treatments were more prone to developing INH resistance (59 [45.03%] versus 13 [0.69%]; p-value<0.001). However, when we compared this factor between high- and low-level INH resistance, we found that the number of previously treated cases was significantly more observed in low-level resistance than high-level resistance (19 [65.51%] versus 40 [39.21%]; p-value=0.019).

 The data were also categorized based on the source of TB infection and the presence of comorbidities (Table 1). The sources revealed that a higher number of patients contracted DR-TB in the workplace compared to those with DS-TB. Specifically in INH-resistant TB, 16 (12.21%) individuals reported workplace infection, 9 (6.87%) had a history of TB among neighbors, 5 (3.81%) reported a family history, while 101 (77.09%) could not recall where they contracted the disease. The study also underscores the impact of comorbidities such as diabetes and anemia on the development of INH resistance, with diabetes being notably more prevalent among DR-TB cases compared to drug-sensitive cases (p-value≤0.001).

**Table 1:** Demographic and associated characteristics of TB cases categorized based on INH resistance levels (high and low) Data are presented for high-level INH resistance cases (n=102), low-level INH resistance cases (n=29), and drug-sensitive TB cases (n=1,862). Variables include age (mean±SD), gender distribution, treatment history, sources of TB infection, and comorbidities. Statistical significance (p-value) is assessed using appropriate tests (chi-square test was used to analyze categorical variables, while independent sample t-test was applied to compare continuous data). NS indicates non-significant differences. Percentages are provided in parentheses where applicable. *p-value<0.001 INH: Isoniazid; NS: Non-significant; SD: Standard deviation; TB: Tuberculosis

Demographic Variables	High-Level INH Resistance (n=102)	Low-Level INH Resistance (n=29)	p-value	INH-Resistant TB (n=131)	Drug-Sensitive TB (n=1,862)	p-value
Age (Mean±SD)	35.93±16.47	38.38±16.42	0.574	36.47±16.42	38.68±18.11	0.175
Age Distribution (Mean±SD)						
Male	40.27±16.28	41.77±15.58	0.926	40.65±16.03	42.49±17.81	0.354
Female	28.63±14.15	27.71±15.30		28.49±14.16*	32.55±16.88	
Gender						
Male	64 (62.75%)	22 (75.86%)	0.268	86 (65.64%)	1145 (63.06%)	NS
Female	38 (37.26%)	7 (24.14%)		45 (34.35%)	714 (38.94%)	NS
Treatment History (Number of Cases [percentage])						
New Cases	62 (60.78%)	10 (34.48%)	0.019	72 (54.96%)	1849 (99.30%)	≤0.001
Previously Treated Cases	40 (39.21%)	19 (65.51%)		59 (45.03%)	13 (0.69%)	
Source of TB Infection						
Unknown Source	77 (74.51%)	24 (82.76%)		101 (77.09%)	1857 (99.73%)	-
History of TB at Workplace	12 (11.64%)	4 (13.79%)	0.466	16 (12.21%)	3 (0.15%)	≤0.001
History of TB in Neighbor	9 (8.82%)	0	-	9 (6.87%)	0	-
History of TB in Relatives	2 (1.96%)	1 (3.44%)	-	3 (2.29%)	0	-
History of TB in Family	2 (1.96%)	0	-	2 (1.52%)	3 (0.16%)	-
Comorbidities						
No Comorbidities	81 (79.41%)	26 (89.66%)	0.529	107 (81.67%)	1852 (99.46%)	-
Diabetes	14 (13.72%)	3 (10.34%)		17 (12.97%)	6 (0.32%)	≤0.001
Anemia	6 (5.88%)	0		6 (4.58%)	4 (0.21%)	-
Surgery	1 (0.98%)	0		1 (0.76%)	0	-

Genetic mutational patterns

In this study, we analyzed the mutational patterns of specific gene linked to INH resistance, which were examined using FL-LPA. High-level INH resistance (*kat*G gene mutation), particularly the *kat*G MUT1 (S315T1) variant at codon 315, was frequently present in 83/131 (63.35%) cases. Another variant, *kat*G MUT1 (S315T2), was also found in 19/131 (14.50%) cases, further indicating the prevalence of mutations at codon 315 in this gene (Table 2). Mutations in the *inh*A promoter region, specifically *inh*A MUT1 (C-15T) at position -15, were observed in 29/131 (22.13%) cases (Table 2). This mutation is recognized as causing low-level INH resistance. This dual mechanism, involving *kat*G and *inh*A mutations, highlights the complexity of INH resistance mechanism in patients with TB. 

**Table 2:** Distribution of katG and inhA mutations contributing to INH resistance INH: Isoniazid

Resistance Gene	Mutation Probe	Codon Analysis	No. of Cases (%)
katG gene mutation (high-level INH resistance)	katG MUT1 (S315T1)	315	83/131 (63.35%)
	katG MUT1 (S315T2)	315	19/131 (14.50%)
inhA gene mutation (low-level INH resistance)	inhA MUT1 (C-15T)	–15	29/131 (22.13%)

Prevalence of INH resistance cases among districts of eastern UP

We have also evaluated the INH resistance prevalence in seven districts linked under IRL. Among the seven districts, Gorakhpur recorded the highest prevalence of INH resistance, i.e., 46/131 (35.11%) cases, followed by Deoria 32/131 (24.43%), Siddharthnagar 22 (16.79%), Kushinagar 19 (14.50%), Sant Kabir Nagar 6 (4.58%), Basti 3 (2.29%), and Maharajganj 3 (2.29%) cases.

**Figure 2:**
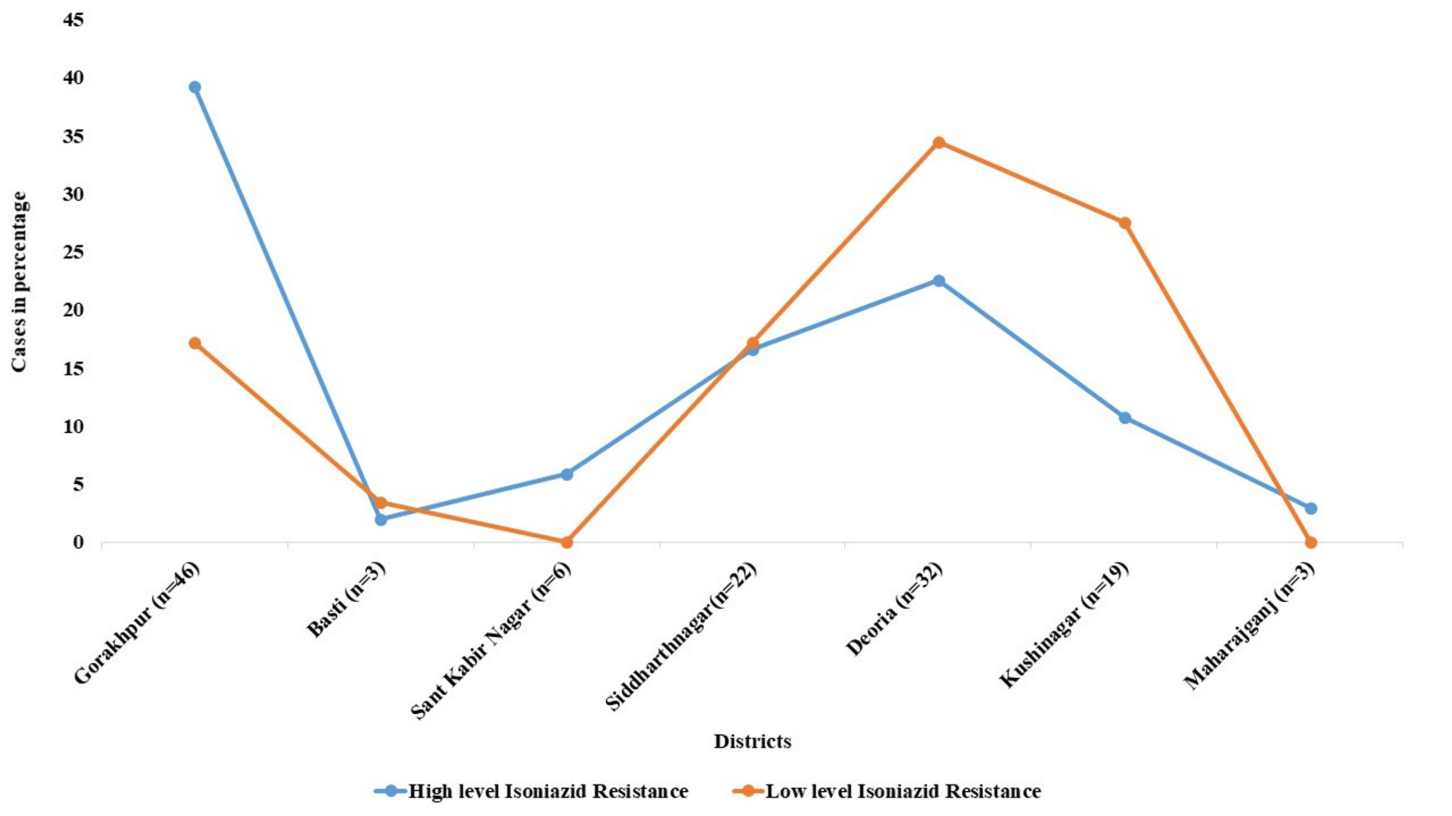
Distribution of INH-resistant cases (n=131) across districts of eastern Uttar Pradesh, categorized by high- and low-level resistance INH: Isoniazid

The prevalence of high- and low-level INH resistance varied across districts linked to the laboratory (Figure 2). Gorakhpur showed the largest percentage of high-level INH resistance, i.e., 40 (39.21%) cases, followed by Deoria 23 (22.54%), Siddharthnagar 17 (12.97%), and Kushinagar 11 (10.78%) cases. Sant Kabir Nagar and Basti, on the other hand, exhibited lower rates of high-level INH resistance.

In cases of low-level INH resistance, 9 (31.03%) cases were detected from Deoria, followed by 8 (27.58%) cases in Kushinagar, 6 (20.68%) cases in Gorakhpur, and 2 (6.8%) cases in Basti. There were not any instances of low-level INH resistance in Sant Kabir Nagar, although Basti had a little larger percentage (3.44%). Kushinagar and Deoria exhibited divergent patterns, with Kushinagar trailing closely behind at 27.58% and Deoria displaying the strongest low-level INH resistance at 34.48% (Figure 2). This shows that in order to stop the development of high-level INH resistance, these areas could need more frequent surveillance and early intervention. Fascinatingly, Siddharthnagar showed equal distribution of INH resistance between low resistance levels (17.24%) and high resistance levels (16.66%).

## Discussion

The results of our study showed different genetic patterns of high and low levels of INH resistance in TB cases from eastern UP, India. Our findings align with some studies while differing from others, highlighting the need to understand resistance mechanisms and regional differences. LPA is proven to be highly accurate for the rapid detection of RMP- and INH-resistant cases, DR-TB cases, or MDR-TB cases. Some studies have already shown that LPA can be used as a reliable test for the early identification of INH-resistant TB cases [15-17]. 

Many studies have reported varying prevalence rates of INH resistance. We reported *kat*G as a common mutation in 85.2% of both MDR-TB and INH monoresistant cases. A study by Mohan et al. also showed a high prevalence of *katG* mutations (96%), similar to our findings [18]. Palani et al. also reported the prevalence of INH monoresistance and MDR-TB at 8.7% and 3.3%, respectively [19]. In our study, the frequency of INH monoresistance was 3.71% and that of MDR-TB was 2.82%, lower than the above study. The differences in INH monoresistance and MDR-TB percentages could be due to variations in study populations, geographical regions, and sample sizes, which influence resistance patterns. Temporal variations and differences in the genetic makeup of *M. tuberculosis* strains may further explain these discrepancies. In a recent study by Mishra et al., the overall burden of MDR-TB was 7.9%, INH monoresistance associated with the inhA gene was 2.8%, and that associated with the *katG* gene was 1.1 % [17]. In contrast, our data revealed a slightly lower overall MDR-TB prevalence of 2.9%, but INH monoresistance due to *kat*G mutations was 5.1%, and due to *inh*A mutations, it was 1.4%, which is quite higher than the above study. The findings of our study on drug resistance patterns in TB align closely with those of Mishra et al., though notable variations exist in the prevalence and distribution of drug resistance mutations. Both studies underscore the importance of molecular diagnostics, particularly the role of the FL-LPA assay, in identifying mutations responsible for resistance to first-line TB drugs [17]. They identify the *kat*G and *inh*A mutations as critical contributors to drug resistance; however, our findings indicate a relatively higher prevalence of *kat*G-related monoresistance compared to Mishra et al.’s study.

Demographically, all studies observed male predominance, with Tilahun et al. reporting 56.3% of male participants and Welekidan et al. noting 63.4% [20,21] of males affected. The age distributions differ slightly, with Tilahun et al.’s cohort study showing that patients were quite younger (mean age: 30.43) compared to our study (mean age: 35.93). This age variation may occur due to differing local epidemiological dynamics, including occupational exposures, comorbidities, and healthcare access patterns [20].

We also found that the history of TB treatment was significantly associated with INH resistance, with 41.18% high-level resistance identified in the previous TB treatment group. This aligns with the findings of Welekidan et al., which highlighted previous treatment as a dominant risk factor for the development of drug resistance. Additionally, the contribution of comorbidities, particularly diabetes, was evident, with a high prevalence (13.72%) among those with high-level resistance. This supports the global understanding of diabetes as a risk factor for DR-TB [21].

In our data, *kat*G S315T mutation was observed in 63.35% of the cases, and it was responsible for high-level INH resistance. This finding aligns with Charoenpak et al., who reported a similar prevalence of *kat*G S315T mutation (70.5%) in mono-H and in MDR-TB isolates (72.7%), emphasizing its consistent role in resistance globally [22]. Another study by Moga et al. reported a *kat*G 315 mutation prevalence of 71% [23].

In contrast, our study found *inh*A mutations (C-15T) in 22.13% of cases, predominantly associated with low-level resistance. This is in line with findings of Raheem et al., who identified 14.6% C-15T mutations [24]. However, another study by Madukaji et al. detected a lower prevalence of *inh*A mutations (11%), suggesting regional variations or differences in strain genetics and study methodologies [25].

The noted differences in high- and low-level INH resistance between districts underscore serious difficulties in TB management. Greater treatment failure rates, longer disease transmission times, and a heavier burden on healthcare systems can all occur in areas with higher resistance levels. Our district-wise analysis revealed significant disparities in resistance levels, with Gorakhpur exhibiting higher rates of high-level resistance and and Deoria showing higher rates of low-level resistance. This geographic variability, supported by findings from Palani et al. in different Indian regions, highlights the importance of local epidemiological surveillance to inform targeted interventions [19].

The major limitation of our study was the use of LPA to detect drug resistance. While LPA is reliable, it has its own boundaries such as its inability to detect mutations that are not present within the targeted genomic regions. The study did not evaluate the clinical significance of genetic mutations by addressing patient treatment responses or outcomes. Another limitation is that the study was undertaken at a single institute, which may limit the generalizability of the findings to other parts of India or globally. Future research could expand the geographical scope and include a more diverse population, which may more accurately reflect the genetic diversity of INH-resistant *M. tuberculosis*, along with the regional differences in drug resistance patterns.

## Conclusions

This study conducted a detailed evaluation of the genetic mutational patterns and distribution of high- and low-level INH resistance in patients with TB in eastern UP. These findings highlight the critical role of *kat*G and *inh*A mutations in drug resistance, with *kat*G mutations being mainly responsible for high-level resistance and *inh*A mutations being associated with low-level resistance. The variation in resistance among districts highlights fundamental problems in TB care, such as delayed diagnosis and treatment failure. A significant proportion of INH-resistant cases was found to be MDR-TB, reinforcing the urgent need for enhanced surveillance and early intervention strategies. Since MDR-TB poses a major challenge to TB control, targeted interventions are essential in high-resistance areas. So, it should be more aggressively addressed in the areas with increased levels of resistance.

The study also underscores the critical need to use molecular diagnostics such as LPA to rapidly detect patterns of resistance. Addressing these challenges requires improved diagnostic facilities, standardized treatment protocols, and integrated comorbidity management, particularly for diabetes. Raising community awareness and increasing routine surveillance are vital in controlling drug-resistant TB, particularly addressing INH resistance through early detection and comprehensive management strategies is crucial to achieving the TB eradication goal.
